# Could the menagerie of the gut microbiome really cure cancer? Hope or hype

**DOI:** 10.1186/s40425-019-0561-9

**Published:** 2019-04-02

**Authors:** Muhammad Bilal Abid

**Affiliations:** 1 Division of Hematology/Oncology, Department of Medicine, Medical College of Wisconsin, Milwaukee, WI USA; 2 Division of Infectious Disease, Department of Medicine, Medical College of Wisconsin, Milwaukee, WI USA

**Keywords:** Immunotherapy, Adoptive cellular therapy, Chimeric antigen receptor (CAR) T-cells, Gut microbiome, Dysbiosis, CRISPR/cas9

## Abstract

The investigational scale of the gut microbiome is expanding rapidly. In 2018, the intersection of gut microbiota and immuno-oncology received much attention. While the impact of gut microbiota on the immune system was already established, the year received an exponential expansion of microbiome’s role in the immunotherapy setting. The microbiome research pipeline is ripe for large-scale, prospective trials. Working knowledge of immune-based cancer treatments, heterogeneity in their responses and resistance mechanisms, relevant immunological and microbiological pathways and potential for gut microbiome in enhancing the responses, is critical.

## Commentary

Evidence continued to mount in 2018 that the gut flora, also called “gut microbiota,” of cancer patients dictates how they respond to a variety of cancer treatments. The bulk of microbiome evidence emerged from immune checkpoint inhibition (ICI) setting in 2018, primarily due to increasing interest in ICI in general. ICI is considered a breakthrough in cancer therapeutics and most recently has been the theme of the Nobel Prize for medicine. While ICI prolongs lives of cancer patients including those who have failed all other types of cancer treatments, it only works in certain types of cancer and that too, in a fraction of patients. Many more lives could be prolonged if the positive responses to ICI were more homogeneous.

The immune system plays a critical role in fighting cancer. Although carcinogenesis is defined by de novo genetic alterations, its sustained progression is dependent upon its ability to evade the host’s immunity [1–7]. With the immuno-oncological revolution, a direct link is now established between tumor sustenance and immunosurveillance failure [5–7]. Cancer cells evade immunity via direct inhibition of CD8+ cytotoxic T-cells (CD8+ T-cells) by employing immune checkpoint pathways, such as programmed cell death –1 (PD-1) and cytotoxic T-lymphocyte antigen-4 (CTLA-4) [2–5]. The ligands for PD-1, PD-L1 and PD-L2‚ are up-regulated in both solid tumors and leukemia/lymphoma [1, 2, 8]. Blockade of the PD-1/PD-L1 and CTLA-4/ligand interactions showed promising activity in multiple solid tumors and hematological malignancies, prompting the approval of PD-1 and CLTA-4 inhibitors [8–13]. These are now used routinely for the treatment of patients with advanced melanoma, non-small-cell lung cancer, head and neck cancer, renal cell cancer, hepatocellular carcinoma, bladder cancer, and Hodgkin’s lymphoma, among several other cancers [9–13].

Although the therapeutic release of immune checkpoints has resulted in unprecedented response rates in patients with a variety of cancers, one-third of patients do not respond. The immunotherapy efficacy also varies considerably depending upon cancer types [2, 3]. Several host’s genetic and immune factors and tumor-related biomarkers have been elucidated that could dictate response [6, 14–16]. Patient’s gut microbiota is being explored as one such determinant of response.

Evolving evidence suggests that diversity and composition of the gut microbiota influences response to immune-based cancer therapies. The initial evidence so far hints towards a relation between certain gut microbial taxa and responses to cancer treatment and survival in both humans and mice, suggesting that its modulation holds substantial therapeutic potential. But the question remains that if the battle against cancer really was situated in the resident taxa, with potential for improved outcomes with simple dietary interventions, then why was not it explored earlier? And, is the existing evidence of manipulating gut microbiome strong enough to be put into clinical practice just yet?

It has been known for more than two decades that gut microbiome interacts with the immune system and impact illnesses directly related to the immune system: autoimmune and infectious diseases. Recent developments in genomic and metagenomic techniques allowed a more robust exploration of the gut microbiome [17, 18]. Its diversity or loss of diversity called “dysbiosis (defined as loss of beneficial microbes, expansion of harmful microbes, and loss of diversity),” and compositional differences have been implicated in obesity, high blood pressure, cardiovascular disease, inflammation, autoimmunity, neurological disorders (also called ‘gut-brain axis’), carcinogenesis (also called ‘onco-microbiome’), and response to vaccines [19–24].

A recent poster presented at the Society for Neuroscience annual meeting was captivating and could be relevant to the interaction between gut fauna and immuno-oncology. The poster showed high-resolution microscopic images of bacteria penetrating and inhabiting the cells of healthy human brains [25]. This hints that the gut microorganisms may not just manipulate health and disease distantly. They may invade and produce desirable or undesirable health outcomes by local invasion of the organ as well. Although its extrapolation towards immunosurveillance in the tumor milieu is still premature, this early cadaveric finding in the brain may carry therapeutic potential in cancers at large, i.e.: local invasion and targeting the enemy inside its home ground, the tumor microenvironment (TME).

The recent genomic exploration of gut taxa allowed stratification of “good or favorable” versus “bad or unfavorable” bacteria in the setting of cancer therapeutics (Table 1). The number of gut microbes and its cumulative genome outnumbers human cells and genome by a considerable fraction [17]. The exact mechanism of how the local immune system in the gut mediates systemic immunity is not known. However, several theories exist. First is that the gut microbiome imparts its tumor-suppressive functions via a variety of proteins and metabolites. Microbial genes encode proteins, some of which are enzymes that generate metabolites. Proteins and/or metabolites could be immune modulators [26, 27]. Specific gut taxa produce several metabolites in the colon via fermentation. These metabolites or short chain fatty acids (SCFAs) then distinctively direct T-cell differentiation patterns, via dendritic cell (DC) activation within the lamina propria of the gut wall. In the mesenteric lymph nodes, DCs then lead to differentiation of naïve T-cells, mainly CD4+ T-cells, into well-characterized T-cell subsets, Th1, Th2, Th17 and forkhead box P3 (Foxp3) + regulatory T-cell (Tregs) [28–30]. These effector T-cells then migrate from mesenteric lymph nodes to the systemic circulation and exert either pro-tumor or anti-tumor effects in TME and systemically. Of these effector T-cells, Th17 are pro-inflammatory and perform an anti-tumor function, whereas Tregs are anti-inflammatory and IL-10 mediates Treg-induced suppression of effector T-cells [28]. Interferon-γ (IFNγ)-production from CD8+ T-cells has also been shown to play a critical anti-tumor role [31–33].

**Table 1:** Major gut microbial taxa and their predominant influence on systemic immunity and response to immunotherapy

Gut Taxa	Immune effect/Treatment response	Study type	Cancer type	Reference
*Predominately positive influence*				
Firmicutes				
*Faecalibacterium prauznitzii*	Boosts effector T-cells and dampens T-regs [38].	Humans	Melanoma	Gopalakrishnan et al. (2018)
*Faecalibacterium spp.*	Increases efficacy of anti-CTLA-4 immunotherapy [44].	Humans	Multiple myeloma	Chaput et al. (2017)
*Eubacterium limosum* *Clostridiales spp.* *Ruminococcaceae spp.* *Phasolarctobacterium spp.*	Boost CD8+ T-cells and enhance anti-PD-1 responses [33].	Cell line	Colorectal Adenocarcinoma Cell line (MC38)	Tanoue et al. (2019)
Fusobacteria				
*Fusobacterium ulcerans* *Fusobacterium varium*	Boost CD8+ T-cells and enhance anti-PD-1 responses [33].	Cell line	MC38	Tanoue et al. (2019)
Verrucomicrobia				
*Akkermansia muciniphilia*	Increase in memory T-cells and decrease in T-regs in the TME [39].	Humans	Epithelial tumors	Routy et al. (2018)
	Increases mucus layer of the gut to prevent lipopolysaccharides absorption [39, 41].	Humans; review	Epithelial tumors	Routy et al. (2018); Routy et al. (2018)
*Mixed influence*				
Bacteroidetes				
*Bacteroides fragilis*	Increases efficacy of anti-CTLA-4 immunotherapy [42].	Human/Animal/Cell line	Epithelial tumors	Vetizou et al. (2015)
	Promotion of T-regs through polysaccharide-A [40].	Humans	N/A	Telesford et al. (2015)
	Higher IL-12 levels in transplant recipients [47].	Animal/Cell line	Cervical cancer	Uribe-Herranz et al. (2018)
*Bacteroides thetaiotaomicron*	Increases efficacy of anti-CTLA-4 and anti-PD-1 immunotherapy [Vetizou et al. showed in anti-CTLA–4 alone; Frankel et al. showed in dual setting] [42, 45].	Humans	Melanoma	Frankel et al. (2017)
*Bacteroides spp.*	Inferior response of anti-CTLA-4 immunotherapy [44].	Humans	Multiple myeloma	Chaput et al. (2017)
Actinobacteria				
*Bifidobacterium longum*	Increases CD8+ T-cells [43, 46].	Animal/Cell line	Melanoma	Sivan et al. (2015)
		Humans	Melanoma	Matson et al. (2018)
*Bifidobacterium bifidum*	Induces naïve T-cell differentiation into T-regs and increases IL-10. Increases the integrity of epithelial barrier [41].	Review	N/A	Routy et al. (2018)
*Predominately negative influence*				
Proteobacteria				
*Enterobacteriaceae*	Inferior response and survival [41].	Review	N/A	Routy et al. (2018)

The second proposed mechanism of how the gut microbiome may modulate anti-tumor immune responses is the cross-reactivity between antigens expressed on the commensal bacteria and neoepitopes found in tumors [34, 35]. However, this mechanism is yet to be explored concretely. But it certainly holds potential as that will provide a link between gut microbial proteins directly shaping the effector T-cell landscape.

Several pre-clinical and clinical studies have highlighted a critical role of the gut microbiota in impacting survival as well as tumor responses to chemotherapy, stem cell transplantation and immunotherapy targeting PD-1, PD-L1, and CTLA-4. The earliest evidence originated from the chemotherapy agent, cyclophosphamide (CYC), one of the most commonly used chemotherapeutic agent for solid tumors and hematologic malignancies as well as for conditioning for the bone marrow transplant (BMT) and for the prevention of graft-versus-host-disease. Part of its therapeutic effect is through the induction of antitumor responses. It became known that CYC alters the composition of gut taxa to stimulate Th17 production which in turn renders the tumor susceptible to CYC [36]. Taur et al. then demonstrated that higher microbial diversity was predictive of decreased mortality in patients who underwent allogeneic hematopoietic stem cell transplantation (alloHCT) [37].

Several human studies in the ICI settings in 2018, performed based on earlier pre-clinical results, have reported positive and reproducible results. Investigators have reported their results in epithelial tumors and hematological malignancies, both in single-agent as well as dual ICI settings. It has been shown that diversity and composition of the gut microbiome mediate response to ICI and improve survival in cancer patients [33, 38–41]. For instance, Gopalakrishnan et al. showed in 43 melanoma patients receiving PD-1 inhibitors that a higher alpha-diversity (within-sample diversity) and relative abundance of bacteria of certain phyla (e.g. *Ruminococcaceae* and *Faecalibacterium* of the *Firmicutes phylum)* are associated with a superior survival and response to ICI. Whereas, a lower diversity of gut microbiome and the abundance of bacteria of certain phyla (e.g. *Bacteroidetes phylum)* are associated with an inferior survival and response to ICI. Mechanistic studies and reverse translational evidence in gnotobiotic mice, germ-free mice that lack intestinal microbiota, corroborate that different groups of bacteria impart distinct immune modulating actions [38]. Routy et al. demonstrated similar findings in a large cohort of 249 patients with diverse epithelial tumors [39].

Several other clinical studies have shown similar results recently [33, 35, 38–46]. Chaput et al. showed a longer survival in 26 multiple myeloma patients treated with anti-CTLA-4, ipilimumab, whose baseline microbiota was enriched with *Faecalibacterium* genus and other *Firmicutes*. In contrast, a high abundance of *Bacteroidetes* was present in subjects with a poor benefit from therapy [44]. Frankel et al. studied the pre-treatment gut microbiota for patients receiving dual ICI for metastatic melanoma. In their findings, the presence of gut taxa belonging to the *Firmicutes* phylum and the abundance of *B. thetaiotaomicron* was associated with efficacy of combined anti-CTLA-4 and anti-PD-1 immunotherapy [45]. Similarly, Matson et al. analyzed 42 patients with metastatic melanoma receiving anti-PD1 therapy alone and showed that 8 species were more abundant in responders, in comparison to non-responders. Fecal microbiota transplants (FMT) into gnotobiotic mice showed that 6 of those identified bacteria, specifically the *Bifidobacterium longum* and *Lactobacillus* species, were associated with slower growth of tumor in mice models [46]. A common pathway among these taxa is DC activation, induction of CD4+ and CD8+ T-cells, an increase in pro-inflammatory Th17 and associated interleukins (e.g. IL-17, IL-12) and a decrease in IL-10 and Tregs [33, 38–46].

Clinical studies in patients receiving concurrent broad-spectrum antibiotics, with immune-based cancer treatments, have shown mixed results [47, 48]. While a few clinical studies have shown that antibiotics during PD-1 inhibition are associated with inferior survival, another study presented at the Society for Immunotherapy of Cancer (SITC) 2018 showed that clinical outcomes were not affected by prior antibiotic use in 111 non-small-cell lung cancer patients, mostly receiving PD-1 inhibition [49]. Since the understanding remains that indiscriminate use of antibiotics globally depletes gut taxa, leads to dysbiosis and hence result in inferior outcomes, further evidence is needed in terms of antibiotic-related impact on gut taxa in cancer patients. Optimal timing of antibiotic administration relative to immune-based therapy also needs to be delineated.

Use of commercially available probiotics is common and has been perceived to be associated with good gut and general health. However, the results of another study presented at the SITC 2018 reported surprising results. In a study conducted in 312 melanoma patients receiving ICI, 42% of patients reported taking probiotics and were found to have a lower diversity of their gut microbiome, in turn, associated with inferior ICI responses and survival [50]. This is a first-of-its-kind result and needs to be investigated further. Favorable gut bacterial ‘signatures’ and ‘biomarkers’ are being identified. The eventual goal will be to have a “designer probiotic,” composed of a rationally-manufactured conglomerate of live bacteria that could be taken safely prior to treatment and patients would be guaranteed an expected level of response.

The data from a few underpowered studies on antibiotics demonstrated mixed results in terms of response and outcomes, as discussed above. Furthermore, SITC study above on the use of probiotics in melanoma patients revealing a lower diversity creates a further contradiction. Hence, the role of pro-, pre- and antibiotics still need to be established via larger, multi-center studies.

The investigational gamut of the gut microbiome is expanding rapidly. A few in vitro studies have already delineated the suppressive role of Tregs in more advanced immune-based therapies, such as adoptive T-cell transfer (ACT). ACTs mainly include chimeric antigen receptor (CAR) T-cells, tumor infiltrating lymphocytes (TILs) and bispecific T-cell engagers (BiTEs). ACT involves the isolation and ex vivo expansion of tumor-specific T-cells and transfusion back to the patient to fight cancer. CAR T-cells are autologous T-cells that are engineered and re-directed towards a tumor-specific antigen [51, 52]. These are a successful modality for patients with refractory B-cell hematological malignancies and are FDA-approved for the treatment of relapsed/refractory acute lymphoblastic leukemia (ALL) and large B-cell lymphoma [53, 54]. TILs are T-cells extracted from patient’s tumors, expanded in vitro and then re-perfused into the patient (reviewed in Rosenberg and Restifo, 2015) [55]. BiTEs recognize 2 different epitopes, 1 for each variable region on the antibody molecule. Blinatumomab is the first FDA-approved BiTE that links T-cells (via CD3) and B-cells (via CD19) to induce tumor cell lysis. Blinatumomab prolonged survival, compared with standard-of-care chemotherapy in adults with relapsed/refractory ALL, in a randomized, open-label, phase-III trial [56].

*Tanoue* et al. further characterized the critical role of IFNγ-expressing CD8+ T-cells in adenocarcinoma, gnotobiotic mice models treated with PD-1 inhibition. The recent study further identified 11 healthy human-associated microbial strains that acted together to inhibit ICI-mediated tumor growth. This therapeutic efficacy was mediated via an increased abundance of IFNγ-expressing CD8+ T-cells. These taxa are under-represented in the general population and predominantly included members from the *Firmicutes* phylum (*Faecalibacterium, Ruminococcacea, Clostridiales,* and *Eubacteria,* etc.)**.** Interestingly, 4 of these strains (3 belonging to the *Firmicutes* phylum), were able to independently induce CD8+ T-cells. Whereas the other 7 strains, belonging to the *Bacteroidetes* phylum, performed CD8+ T-cells induction only in conjunction with the other 4 strains. Majority of the remaining strains, that were studied and were found to lack tumor-suppressive effect, belonged to the *Bacteroidetes* phylum [33]. Although studies have shown mixed results in identifying influential strains so far (summarized in Table 1), *Tanoue* et al. shared critical finding towards establishing a rationally-designed microbial product for future trials. A CD8+ T-cells-based therapeutic design will have broader application towards all immune-based, anti-tumor treatment strategies.

The role of antibiotics has been studied in the ACT setting as well. In a study on mouse models, Uribe-Herranz et al. showed that vancomycin depleted *Bacteroides spp*. and augmented the function of adoptively transferred antitumor T-cells, in an IL-12–dependent manner, which is also responsible for an increased abundance of effector T-cells in the TME. To demonstrate a causal effect in humans, they further showed higher levels of IL-12 in those alloHCT patients who had received oral vancomycin [47]. However, the *Bacteroides*-induced suppression of ACTs demonstrated by Uribe-Herranz et al. was in contradiction with the study results of Vétizou et al. who had earlier shown that the *Bacteroides* species, specifically *B. fragilis* and *B. thetaiotaomicron*, promote the efficacy of CTLA-4 blockade in mice [42]. Kuczma et al. studied the impact of antibiotics in mice in the ACT setting and showed that antibiotics dampened CYC-induced endogenous T-cell responses. Interestingly, long-term antibiotics had no impact on the efficacy of CD19+ CAR T-cells used for lymphoma although it impacted the long-term persistence of CAR T-cells [48].

With an established ability of the gut microbiome in suppressing Tregs, large, prospective studies are being conducted in the ICI, ACT and CAR T-cells settings. Some critical trials that are currently underway include: modification of gut microbiome by dietary intervention (non-absorbable oligosaccharides contained in potato starch) in patients undergoing BMT at the University of Michigan (NCT02763033), FMT from healthy donors in patients undergoing BMT to study survival, post-BMT complications and graft-versus-host-disease incidence at the Massachusetts General Hospital (NCT03720392), concurrent FMT with immunotherapy at the University of Pittsburg (NCT03341143), administration of a rationally-designed bacterial consortia along with immunotherapy (NCT03595683), and transplantation of taxa from responders into non-responders. For instance, a phase-I trial is currently recruiting in Israel in metastatic melanoma patients to study safety and response to FMT from immunotherapy responders to refractory patients (NCT03353402). Groups at MD Anderson and Memorial Sloan Kettering Cancer Centers USA as well as in France are also actively studying, in collaboration with the industry, the potential impact of certain taxa on treatment responses and patients’ survival. These studies will be directed toward averting resistance mechanisms to the novel therapies explored thus far.

The immunological evidence behind gut microbiome’s potential to modulate responses to cancer treatments is strong. It is a matter of time that we will be able to show that the gut microbiome modulation works in large, multi-center, prospective trials. Probiotics, narrow-spectrum antibiotics, non-absorbable oligosaccharides contained in potato starch or even a certain diet, fecal transplant from healthy donors, are all potential interventional strategies. These could be employed to strategically modify microbiota, enhance responses to cancer treatment and prolong lives. We are far from that. But we are aware that dysbiosis can increase the representation of deleterious microbiota that produces harmful metabolites and antigens and lead to maladaptive immune responses. Strategically averting gut dysbiosis, preventing alpha diversity crash during treatment, and maintaining desirable taxa are needed to augment response to cancer treatment.

Programmable DNA cutters are being utilized to knockout inhibitory proteins. For instance, utilizing CRISPR/cas9-based gene-editing showed an enhanced efficacy of CAR T-cells in the tumor mice model. Ren et al. manufactured potent, universal CAR T-cells with knockout inhibitory ligands, including PD-1, endogenous TCR, and β-2 microglobulin, utilizing CRISPR/cas9 multiplex gene-editing [57]. Rupp et al. generated PD-1 deficient CD19+ CAR T-cells via a similar mechanism [58]. Microbiome manipulation holds at least similar potential, if not superior, in enhancing treatment responses to ICI and tumor-antigen directed engineered T-cells as that of more sophisticated genome-editing technology.

The field of “onco-microbiome” is evolving. Driven by the era of precision oncology, it is likely to draw greater interest and funding. The impact of gut microbiome on immune-based cancer therapeutics will be a breakthrough in terms of improving patients’ outcomes and the field is certainly ripe to live up to its hype.

## Abbreviations

ACT: Adoptive T-cell transfer
AlloHCT: Allogeneic hematopoietic stem cell transplantation
CAR T-cells: Chimeric antigen receptor T-cells
CRISPR: Clustered regularly interspaced short palindromic repeats
CYC: Cyclophosphamide
DC: Dendritic cell
ICI: Immune checkpoint inhibition
mLN: Mesenteric lymph nodes
SCFA: Short chain fatty acid
TME: Tumor microenvironment
Tregs: Regulatory T-cells
